# Supplementary material for: Hydrophobic interactions dominate the recognition of a KRAS G12V neoantigen
Source: Nat Commun. 2023 Aug 21;14:5063. doi: 10.1038/s41467-023-40821-w (PMC10442379; doi:10.1038/s41467-023-40821-w)
Supplement: Supplementary file 1 — Supplementary Information [file 41467_2023_40821_MOESM1_ESM.pdf]

Hydrophobic interactions dominate the recognition of a KRAS G12V neoantigen

Katharine M. Wright[1,2,3,4,19], Sarah R. DiNapoli[2,5,6,19], Michelle S. Miller[1,2,3,7,19], P. Aitana Azurmendi[1,2,3], Xiaowei Zhao[8], Zhiheng Yu[8], Mayukh Chakrabarti[1], WuXian Shi[9,10], Jacqueline Douglass[2,5,6], Michael S. Hwang[2,5,6], Emily Han-Chung Hsiue[2,5,6,11], Brian J Mog[2,5,6,12], Alexander H Pearlman[2,5,6], Suman Paul[5,6,13,14], Maximilian F Konig[2,5,6,15], Drew M Pardoll[3,13] Chetan Bettegowda[5,6,13,16], Nickolas Papadopoulos[5,6,13,17], Kenneth W Kinzler[3,5,6,13], Bert Vogelstein[2,3,5,6,13,17], Shibin Zhou[3,5,6,13,20], Sandra B. Gabelli[1,3,4,13,18,20]

Affiliations:

[1] Department of Biophysics and Biophysical Chemistry, The Johns Hopkins School of Medicine, Baltimore, MD 21205, USA

[2] Howard Hughes Medical Institute, Chevy Chase, MD 20815, USA.

[3] Bloomberg~Kimmel Institute for Cancer Immunotherapy, Sidney Kimmel Comprehensive Cancer Center, Baltimore, MD 21287, USA.

[5] Ludwig Center, Sidney Kimmel Comprehensive Cancer Center, Johns Hopkins University School of Medicine, Baltimore, MD 21287, USA.

[6] Lustgarten Pancreatic Cancer Research Laboratory, Sidney Kimmel Comprehensive Cancer Center, Johns Hopkins University School of Medicine, Baltimore, MD 21287, USA.

[8] Janelia Research Campus, HHMI,19700 Helix Drive, Ashburn, Virgina 20147

[9] Energy & Photon Sciences Directorate, Brookhaven National Laboratory, Upton, NY 11973, USA.

[10] Case Center for Synchrotron Biosciences, Case Western Reserve University, Cleveland, OH 44106, USA.

[12] Department of Biomedical Engineering, Johns Hopkins University, Baltimore, MD 21218, USA.

[13] Department of Oncology, Johns Hopkins University School of Medicine, Baltimore, MD 21287, USA.

[14] Division of Hematologic Malignancies and Bone Marrow Transplantation, Johns Hopkins University School of Medicine, Baltimore, MD 21287, USA.

[15] Division of Rheumatology, Department of Medicine, Johns Hopkins University School of Medicine, Baltimore, MD 21224, USA.

[16] Department of Neurosurgery, Johns Hopkins University School of Medicine, MD 21205, USA.

[17] Department of Pathology, Johns Hopkins University School of Medicine, Baltimore, MD 21205, USA.

[18] Department of Medicine, Johns Hopkins University School of Medicine, Baltimore, MD 21205, USA.

[4] Present address: Discovery Chemistry, Protein and Structural Chemistry, Merck & Co, Inc. West Point, PA, 19846, USA.

[7] Present address: Walter and Eliza Hall Institute, Parkville, Vic, 3052, Australia

[11] Present address: Novartis Institutes for BioMedical Research, 250 Massachusetts Ave, Cambridge, MA 02139

[19] These authors contributed equally: Katharine M. Wright, Sarah R. DiNapoli, Michelle S. Miller

[20] Corresponding authors, gabelli@jhmi.edu; sbzhou@jhmi.edu

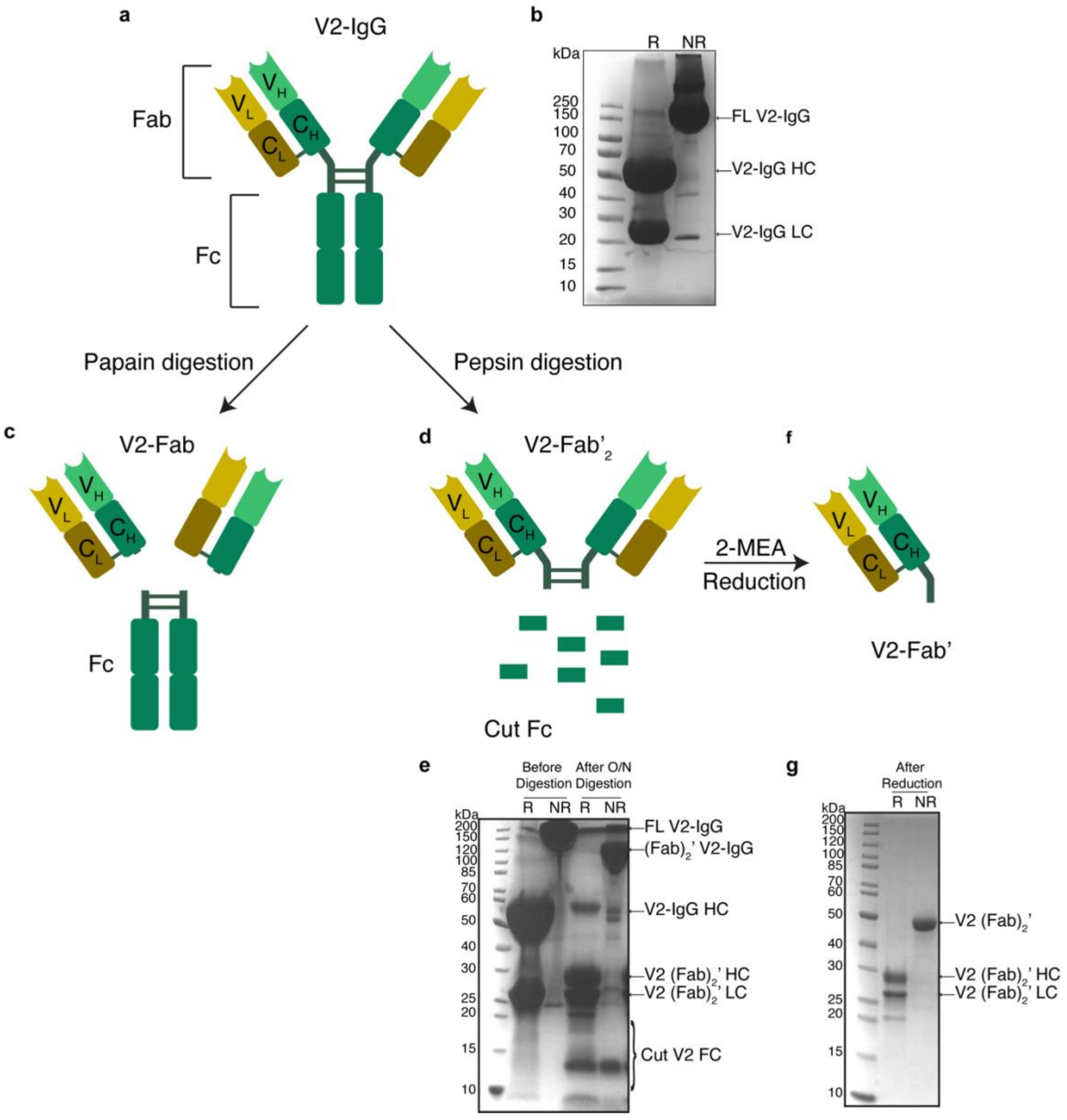

Supplementary Figure 1: Enzymatic cleavage of V2-IgG. a Depiction of the full-length V2-IgG. **b** Coomassie-stained gradient SDS-PAGE gel of full-length V2-IgG. R, reducing 2X loading buffer; NR, non-reducing 2X loading buffer; HC, heavy chain; LC, light chain. N > 5 independent experiments. **c** Scheme depicting the enzymatic cleavage of the V2-IgG with papain, which results in V2-Fab and Fc molecules. **d** Scheme depicting the enzymatic cleavage of the V2-IgG with pepsin, which generates V2-(Fab)$_2$'. **e** Coomassie-stained gradient SDS-PAGE gel of

overnight pepsin digestion of V2-IgG. R, reducing 2X loading buffer; NR, non-reducing 2X loading buffer; HC, heavy chain; LC, light chain. N > 5 independent experiments. **f** Scheme depicting reduction with 2-MEA of the V2-$(Fab)_2$' to V2-Fab'. **g** Coomassie-stained gradient SDS-PAGE gel of 2-MEA reduction of V2-$(Fab)_2$'. R, reducing 2X loading buffer; NR, non-reducing 2X loading buffer; HC, heavy chain; LC, light chain. N > 5 independent experiments.

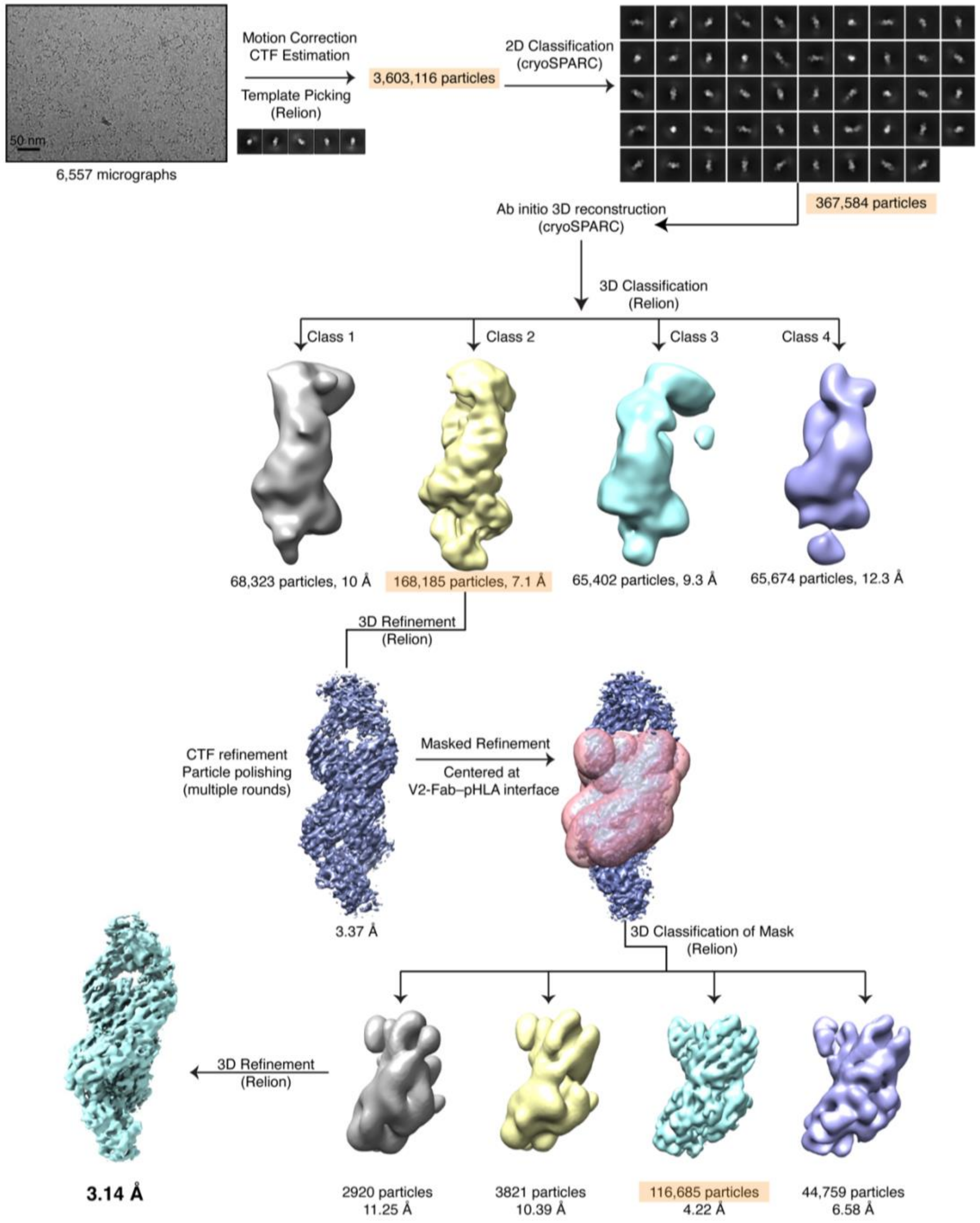

Supplementary Figure 2: Cryo-EM reconstruction of V2-Fab/KRAS^{G12V}-HLA-A*03:01.

Flowchart for the cryo-EM data processing. A value of T=4 was used for both 3D classification steps. The final model has an average resolution of 3.14 Å. All the images in this figure were rendered with ChimeraX.

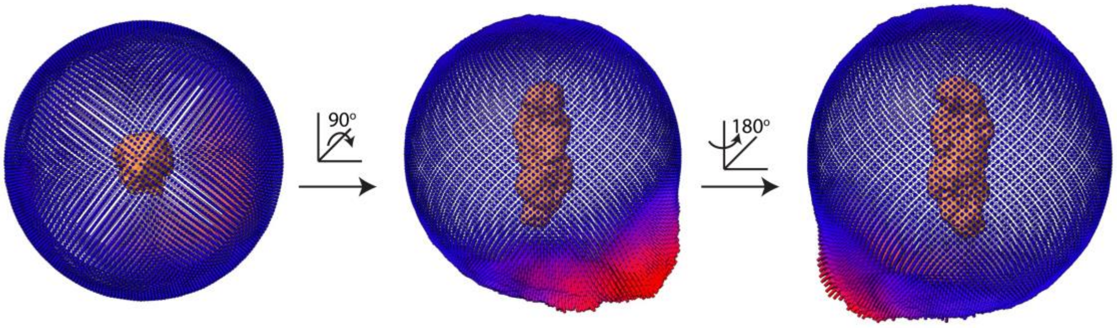

Supplementary Figure 3: Euler angle distribution plots. Euler angle distribution plots show good angular coverage with a slight preferred orientation.

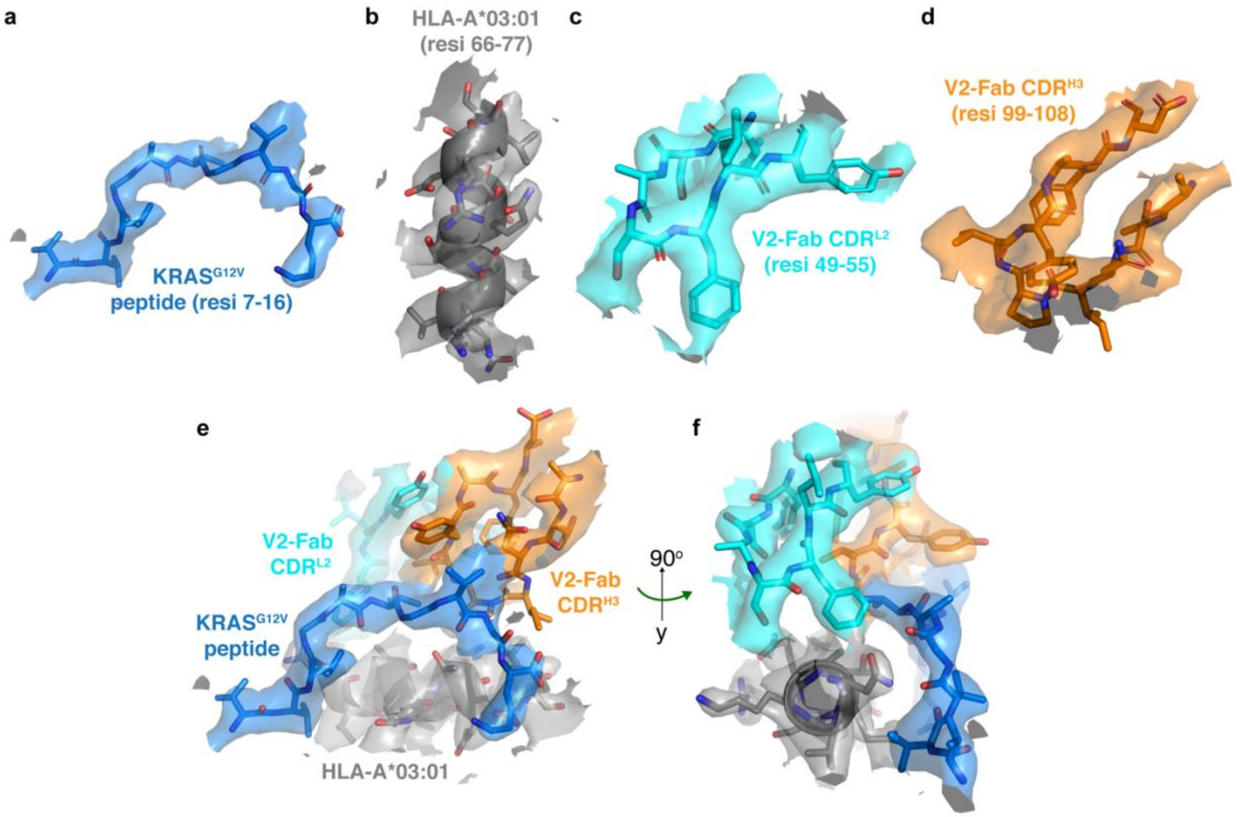

Supplementary Figure 4: Segments of EM-map of the V2-Fab/KRAS^{G12V}-HLA-A*03:01 binding interface. For each panel, the molecular model is shown in stick representation, and the cryo-EM map as a transparent surface colored by chain at a threshold level of 0.01914. **a** Atomic model of the KRAS^{G12V} peptide residues 7-16 in the cryo-EM density map. **b** As in A, for the HLA-A*03:01 α1, residues 66-77. **c** As in A, for V2-Fab CDR^{L2}, residues 49-55. **d** As in A, for the V2-Fab CDR^{H3}, residues 99-108. **e** Atomic model of the KRAS^{G12V} peptide (A), HLA-A*03:01 α1 (B), V2-Fab CDR^{L2} (C), and V2-Fab CDR^{H3} (D) as seen in the cryo-EM map of the V2-Fab/KRAS^{G12V}-HLA-A*03:01 structure. **f** View of the cryo-EM map at 90° rotation to that shown in E.

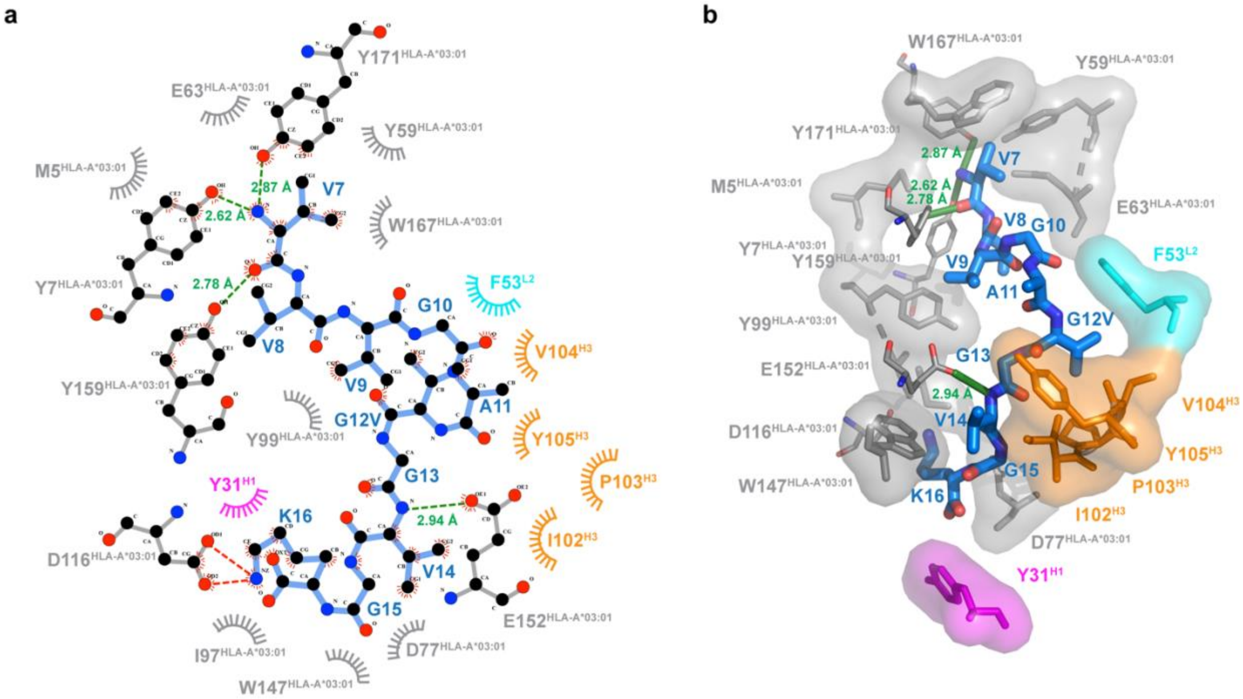

Supplementary Figure 5: LigPlot diagrams showing the HLA-A*03:01 and V2-Fab interactions with the KRAS^{G12V} peptide. a LigPlot diagram of the V2-Fab bound to KRAS^{G12V}/HLA-A*03:01. The KRAS^{G12V} peptide (ligand) is shown in blue sticks. Hydrogen bonds are shown in green lines and hydrophobic interactions are highlighted by the half circle spokes pointed toward the peptide; colored according to CDR. **b** Pymol representation of the LigPlot in A. Hydrogen bonds are shown in green lines. Surface representation is shown for all residues involved in hydrophobic interactions with the KRAS^{G12V} peptide. All residues are colored according to HLA-A*03:01 (gray), peptide (blue) and CDRs.

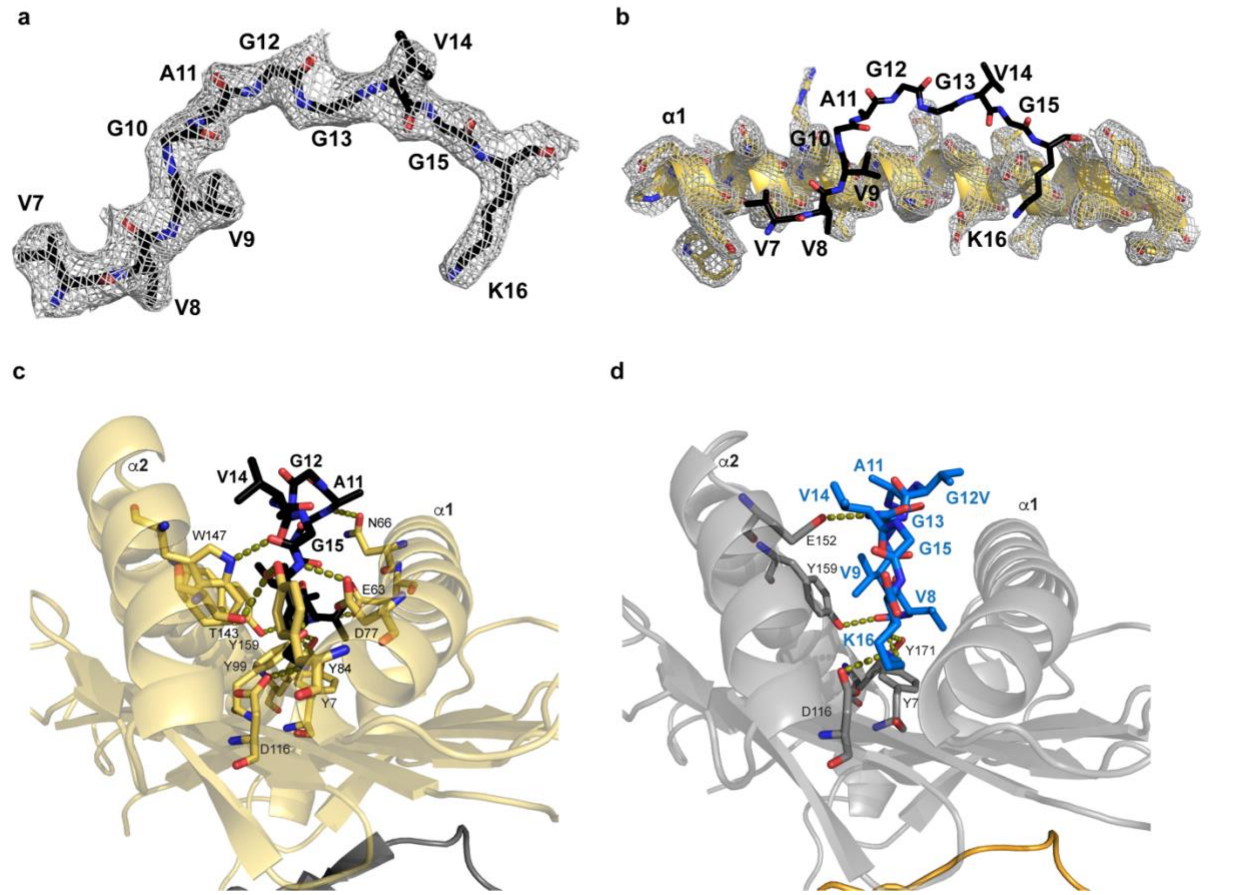

Supplementary Figure 6: The pHLA binding groove is well-resolved. a 2mFo-DFc electron density map of the KRAS^{WT} peptide (σ=1). **b** 2mFo-DFc electron density map of the HLA-A*03:01 binding groove, highlighting the α1 of the HLA. The KRAS^{WT} peptide is shown in black as sticks (σ=1). **c** Perpendicular view of the KRAS^{WT} peptide binding cleft as shown in Fig. 3B. **d** Perpendicular view of the $\text{KRAS}^{\text{G12V}}$ peptide binding cleft as shown in Fig. 3D.

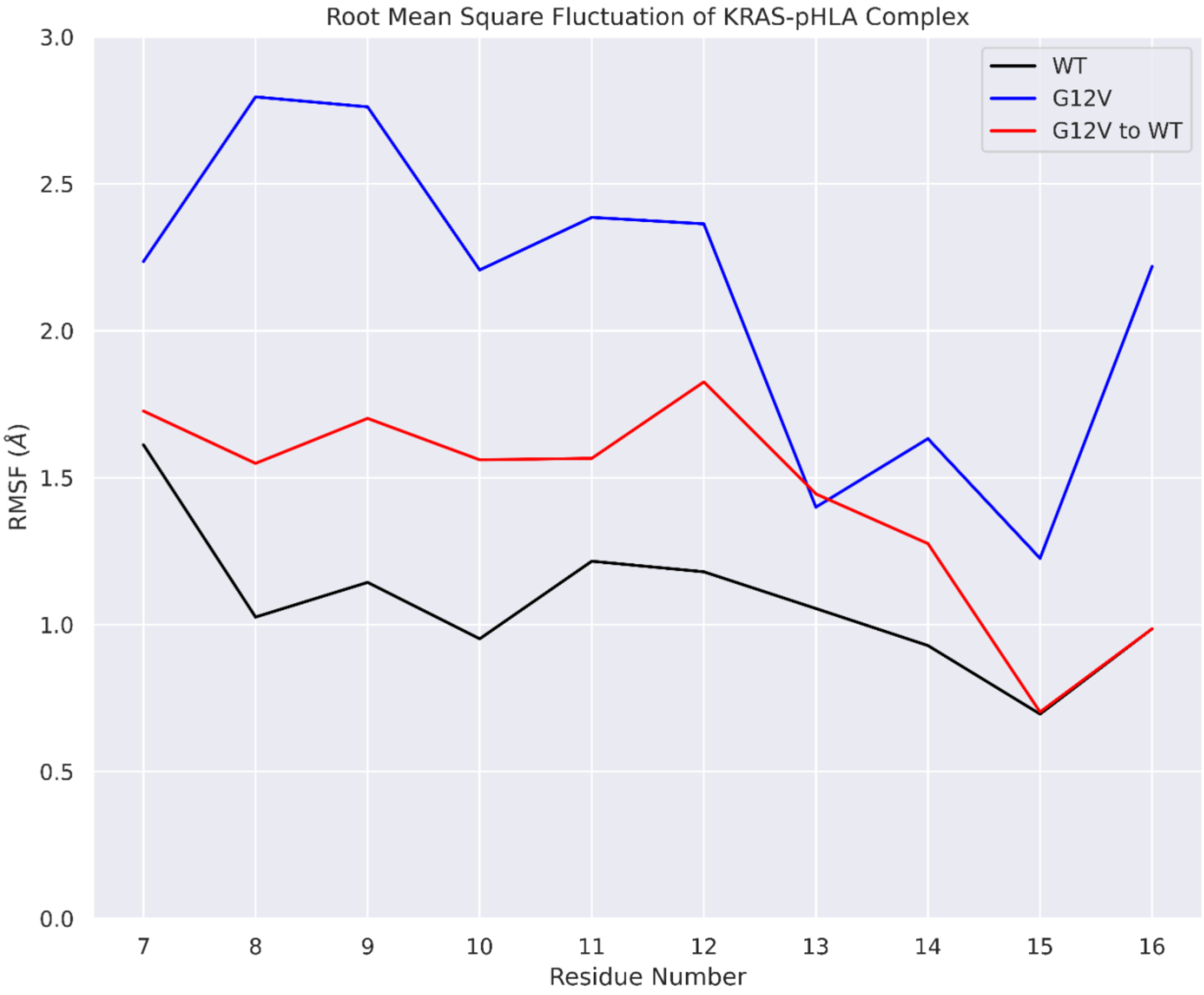

Supplementary Figure 7: Root Mean Square Fluctuation (RMSF) of KRAS-pHLA complexes. The residue fluctuations (in Ångströms) of the $KRAS^{WT}$ peptide (black), $KRAS^{G12V}$ peptide (blue), and $KRAS^{G12V}$ peptide reverted to $KRAS^{WT}$ (red), each complexed with HLA-A*03:01, are shown based on an aggregated molecular simulation sampling time of 1.02 µs for each system.

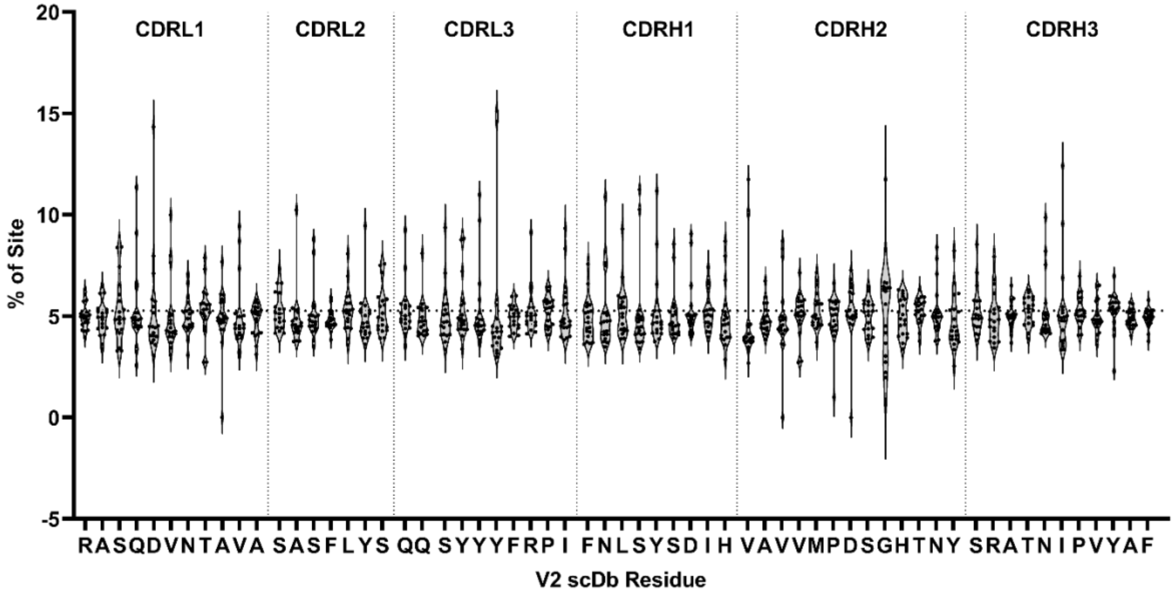

Supplementary Figure 8: Affinity Maturation Library Diversity after Synthesis. After synthesis, Twist Bioscience used next generation sequencing to quantify the proportion of total reads per site for each variant at that site. Individual variant frequencies are plotted by position. If all variants are represented at an equal frequency for a given site, each variant should account for 5.263% of total reads (1/19, dotted line). Three variants were not detected by NGS: A32M (CDRL1), V50M, and D54W (both CDRH2). The overall average frequency was 5.092 ± 1.394% (n = 1159 variants sequenced).

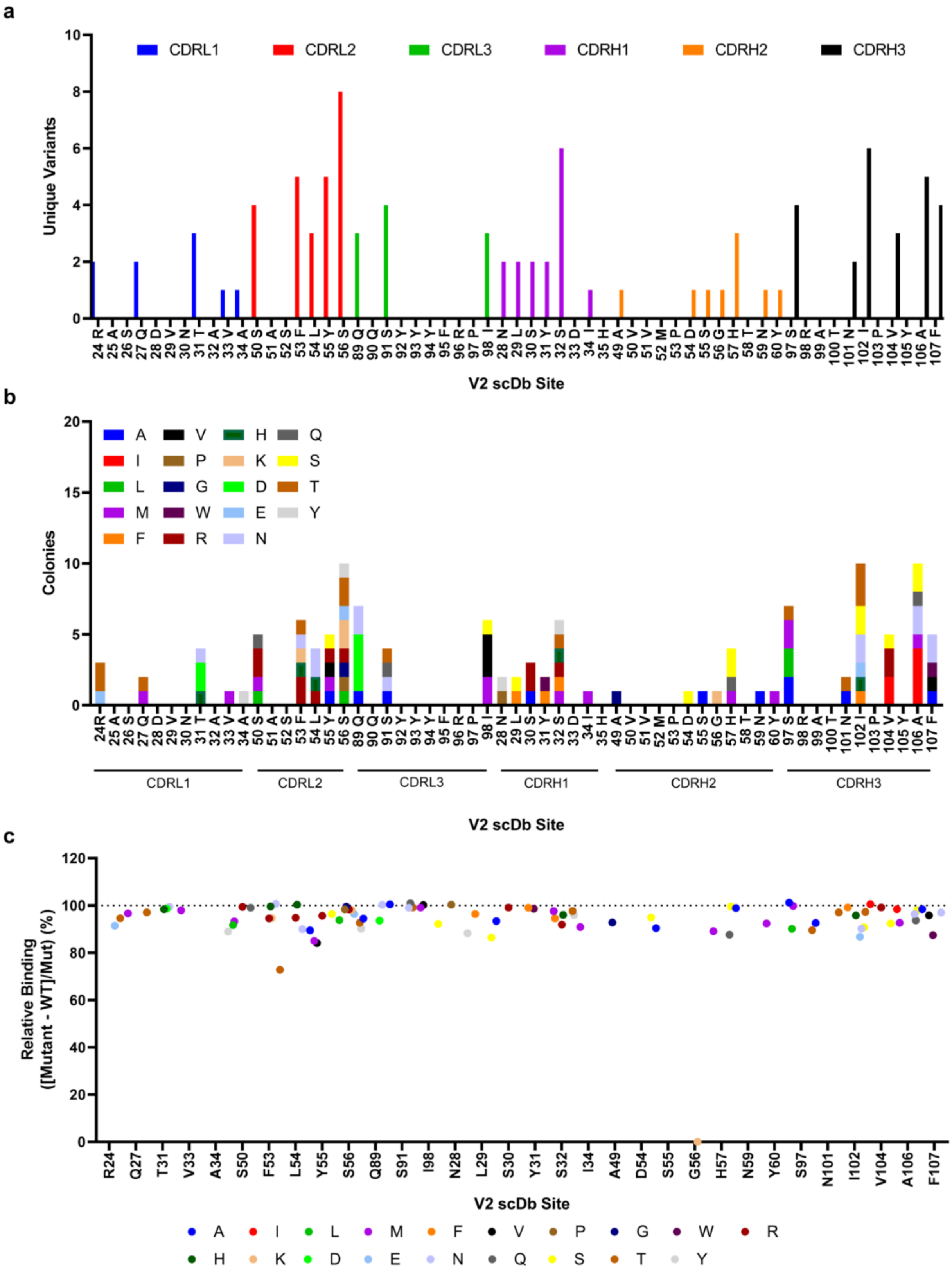
a
Unique Variants
CDRL1
CDRL2
CDRL3
CDRH1
CDRH2
CDRH3
V2 scDb Site
b
Colonies
A V H Q
I P K S
L G D T
M W E Y
F R N
CDRL1
CDRL2
CDRL3
CDRH1
CDRH2
CDRH3
V2 scDb Site
c
Relative Binding ([Mutant - WT]/Mut) (%)
V2 scDb Site
A I L M F V P G W R
H K D E N Q S T Y

Supplementary Figure 9: Monoclonal Phage Characterization after Panning. Monoclonal phage colonies were picked and identified by Sanger sequencing after rounds 4 and 5 of panning. Site in the light and heavy chains, and starting amino acid, are listed on the X axis. **a** The number of unique variants per site is displayed across the 61 sites included in the affinity maturation library. A total of 92 unique variants were present in 123 picked colonies. **b** The number of colonies per variant is displayed for each site in the affinity maturation library. **c** Monoclonal phage specificity for the $KRAS^{G12V}$–HLA-A*3:01 monomer was compared to the $KRAS^{WT}$–HLA-A*3:01 monomer by ELISA using the A_{450} value with an A_{540} correction. Relative binding was calculated as $(Abs_{mutant} – Abs_{wild\ type})/ Abs_{mutant} \times 100\%$.

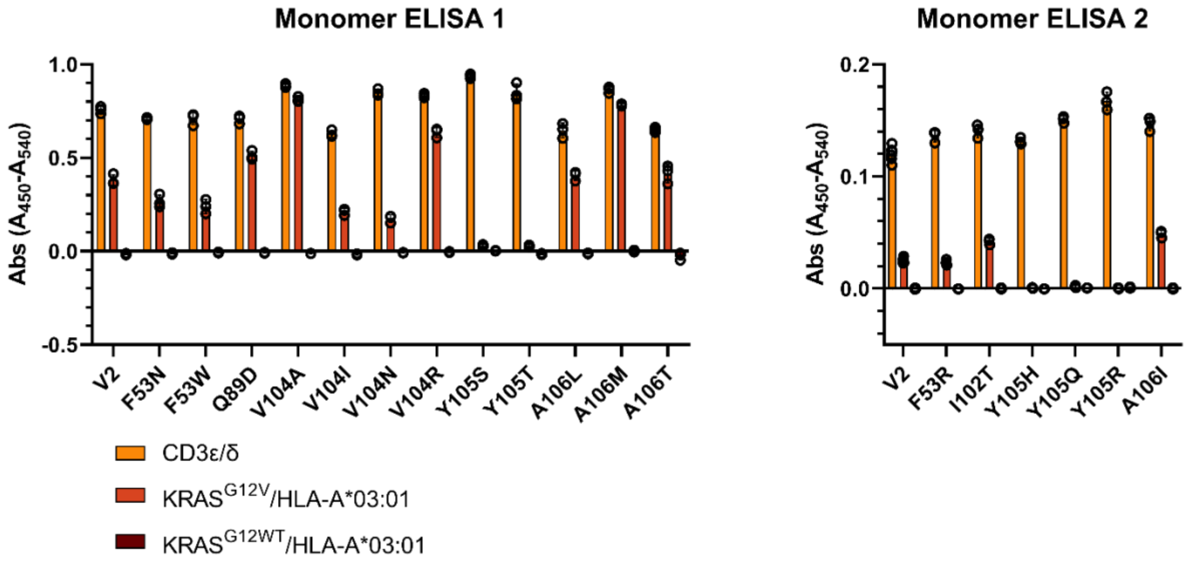

Supplementary Figure 10: scDb binding to CD3ε/CD3δ heterodimer, KRAS^{G12}V/A3, and KRAS^{WT}/A3. scDb at 200 ng/ml was incubated on ELISA plates coated with CD3ε/CD3δ heterodimer (light orange), KRAS^{G12V}–HLA-A*03:01 (dark orange), and KRAS^{WT}–HLA-A*03:01 (dark red) for 1 h, detected by Protein L and anti-Protein L-HRP. A_{450} values with an A_{540} correction are reported for each scDb. Plotted data indicate mean ± SD, n = 3 biologically independent samples per condition from one experiment.

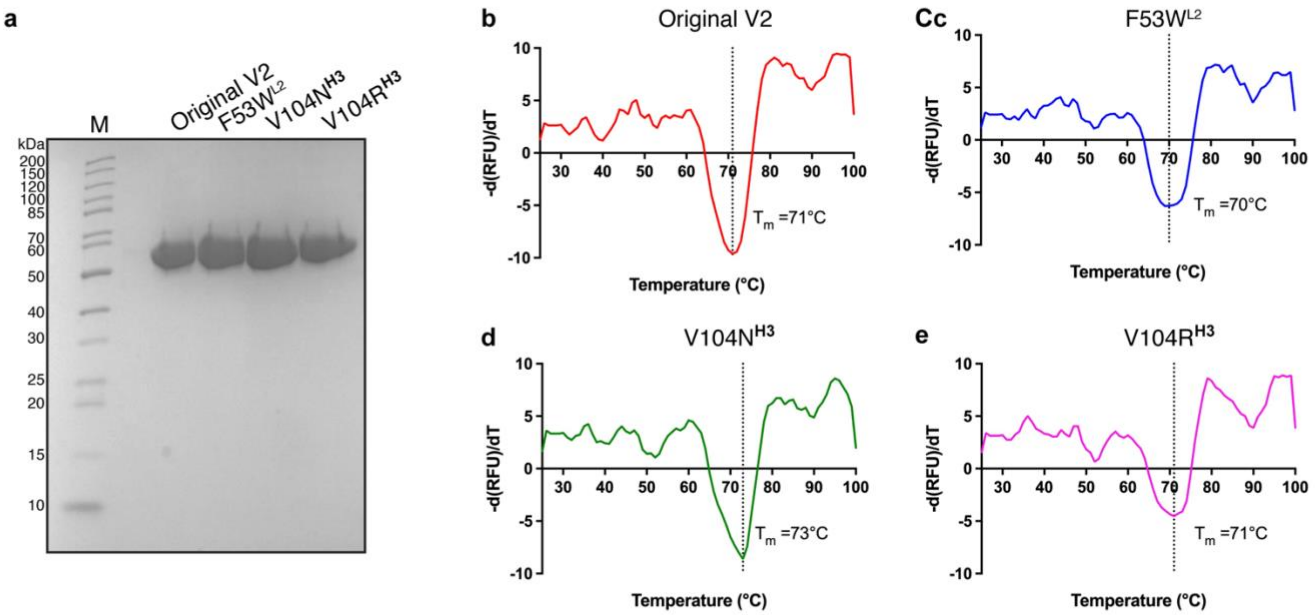

Supplementary Figure 11: Purification and Differential Scanning Fluorimetry (DSF) of the V2 scDb variants. a SDS-PAGE of the original V2 and variant scDbs, running at the expected molecular weight of 55 kDa, lacking contaminants. N > 3 independent experiments. **b** Differential scanning fluorimetry analysis of original V2 scDb (red). The negative derivative of relative fluorescence unit (RFU) vs temperature is shown. The melting temperatures are labeled and correspond to the minimum peak of each first derivative. The dotted line highlights the temperature that aligns with the minimum -d(RFU)/dT. **c** DSF graph of F53W^{L2} V2 scDb (blue). **d** DSF graph of V104N^{H3} V2 scDb (green). **e** DSF graph of V104R^{H3} V2 scDb (magenta).

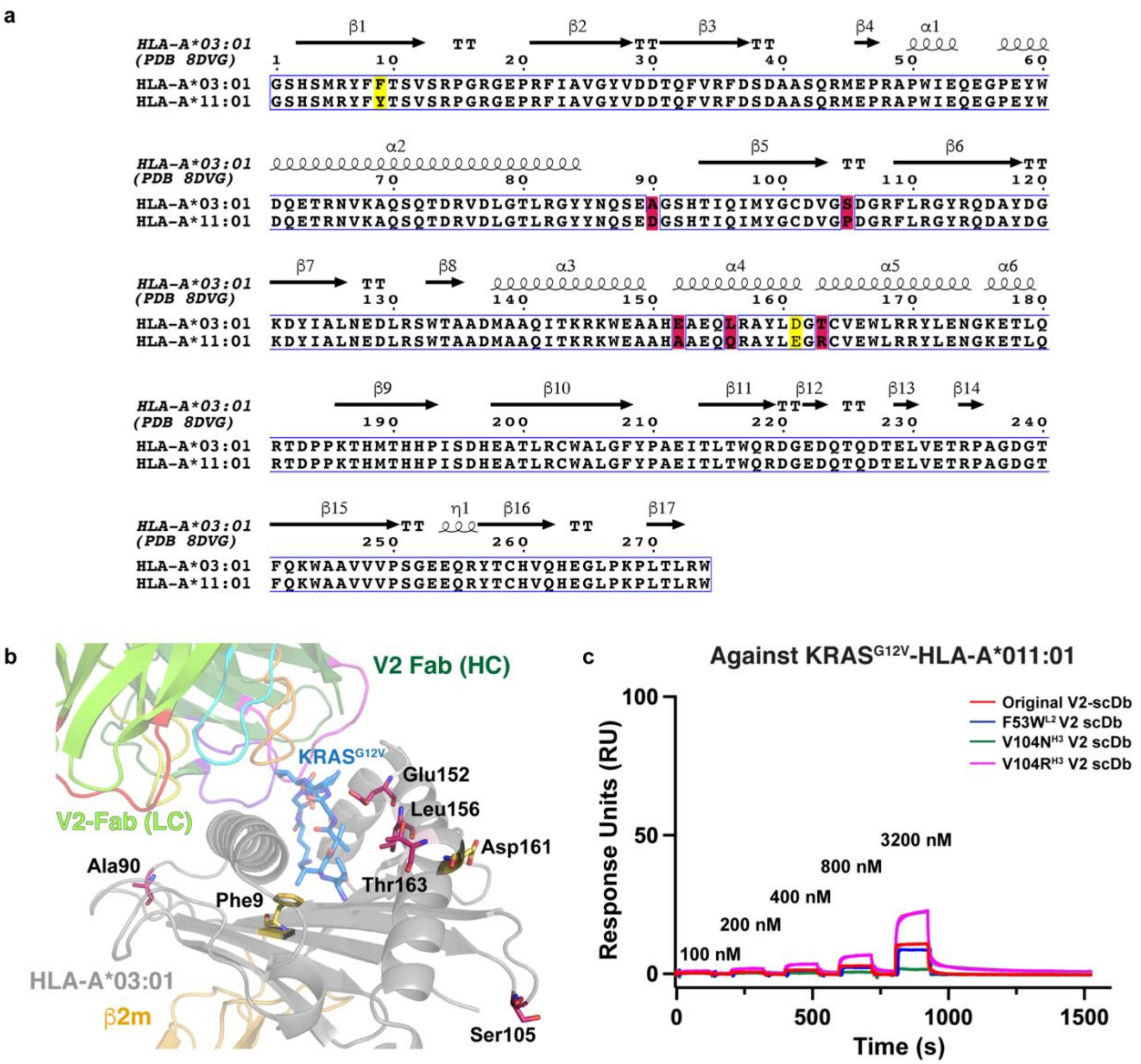

Supplementary Figure 12: Binding kinetics of the V2 scDb against KRAS^{G12V}-HLA-A*11:01. a Sequence alignment of HLA-A*03:01 and HLA-A*11:01. Identical amino acids are in black with background in white, similar amino acids are in black with yellow background, and different amino acids are in black with maroon background. The KRASWT-HLA-A*03:01 structure (PDB ID 8DVG) was used for secondary structure elements; shown as arrows (β-strands) and springs (α-helices). **b** Zoomed-in view of the V2 Fab/KRAS^{G12V}-HLA-A*03:01

complex, focusing on the peptide binding groove. The location of the seven residues that differ in HLA-A*11:01 are shown in sticks and colored as in A. **c** Original V2 and variant scDbs binding to KRAS^{G12V}-HLA-A*11:01 were evaluated by single-cycle kinetics SPR. V2 scDb (red) bound to pHLA with two state binding kinetics and a K_D of 12.1 µM. F53W^{L2} (blue) and V104N^{H3} (green) did not bind the pHLA. V104R^{H3} scDb (magenta) bound to pHLA with two state binding kinetics and a K_D of 7.8 µM. All sensorgrams are a representative experiment of n =3 independent experiments.

Supplementary Table 1. Contacts between V2-Fab and KRAS^{G12V}-HLA-A*03:01.

Contacts/bonds were calculated with a 4 Å cutoff, which includes hydrogen bonds and van der Waals interactions, using CONTACT in the CCP4 suite (49). BSA, buried surface area; HC, heavy chain; LC, light chain.

	V2-Fab to KRAS^{G12V}/HLA-A*03:01
Crossing Angle	35°
Incident Angle	24°
Total contacts	175
Peptide residues interacting with V2-Fab	6, 7, 8, 9, 10
Peptide contacts	26 (15.5%)
Bonds from HC	21
Hydrogen Bonds	0
Hydrophobic	21
Bonds from LC	5
Hydrogen Bonds	0
Hydrophobic	5
HLA contacts	149
Bonds from HC	98
Bonds from LC	51
BSA (Å^2)	
BSA total	1388
BSA HC pep	197.4
BSA LC pep	64.7
BSA HC HLA	664.5
BSA LC HLA	461.8
CDRs interacting with peptide	L2, H1, H3
CDRs interacting with site of mutation	L2, H1, H3

Supplementary Table 2. Atom level contacts between V2-Fab CDRs and KRAS^{G12V} peptide. Contacts were calculated with a 4 Å cutoff, which includes hydrogen bonds and van der Waals interactions, using CONTACT in the CCP4 suite (49).

V2-Fab Light Chain

CDR Number	CDR Residue and Atom	KRAS^{G12V} Residue and Atom	Distance (Å)
CDRL2	Phe53 CD1	Val12 CG2	3.84
	Phe53 CB	Val12 CG2	3.86
	Phe53 CG	Val12 CG2	3.83
	Phe53 CZ	Gly10 O	3.14

V2-Fab Heavy Chain

CDR Number	Amino Acid Residue and Atom	KRAS^{G12V} Residue and Atom	Distance (Å)
CDRH1	Tyr31 CD2	Lys16 O	3.90
	Tyr31 CE2	Lys16 OXT	3.74
CDRH3	Asn101 O	Gly15 CA	3.98
	Ile102 CG1	Gly15 N	3.63
	Ile102 CA	Gly15 N	3.88
	Ile102 CD1	Val14 CG2	3.54
	Ile102 CG1	Val14CG2	3.67
	Ile102 CD1	Val14 CG1	3.92
	Ile102 CG1	Val14 CA	3.28
		Val14 C	4.00
		Val14 CB	3.70
		Val14 CG1	3.59
	Ile102 CG2	Val12 CG1	3.91
	Ile102 CG2	Gly13 O	3.22
	Pro103 CD	Gly13 O	3.89
	Pro103 CG	Val12 CG1	3.69
	Val104 N	Val12 CG1	3.69
	Val104 CB	Val12 CG1	3.83
	Val104 CG2	Val12 CG1	3.72
	Val104 CB	Val12 CG2	3.96
	Val104 CG2	Va12 CG2	3.77
	Tyr105 CE2	Val12 O	3.75

Supplementary Table 3. V2 Variants Tested. 18 variants in CDRL2, CDRL3, and CDRH3 were selected for functional characterization as scDb based on the cryo-EM crystal structure of the V2 in complex with the KRAS^{G12V}-HLA-A3 monomer.

Chain	CDR	Residue	Variant	% of Monoclonal Phage colonies*
Light	CDRL2	53	F53N	0.8
			F53R	1.6
			F53W	0
	CDRL3	89	Q89D	3.3
Heavy	CDRH3	102	I102T	2.4
		104	V104A	0
			V104I	1.6
			V104N	0
			V104R	1.6
		105	Y105H	0
			Y105Q	0
			Y105R	0
			Y105S	0
			Y105T	0
		106	A106I	3.3
			A106L	0
			A106M	0
			A106T	0

*Percent of 123 unique monoclonal phage colonies sequenced after rounds 4 and 5 of panning.

Supplementary Table 4. Kinetics and affinity, as determined by SPR, of the V2-scDb against mutant and WT KRAS peptide bound to pHLA-A3 and pHLA-A11. Nd, not determined; NB, no binding.

	**$KRAS^{G12V}$-HLA-A*03:01**							**$KRAS^{WT}$-HLA-A*03:01**						
	$k_{on}1$ (1/Ms)	$k_{off}1$ (1/s)	$k_{on}2$ (1/s)	$k_{off}2$ (1/s)	K_D (nM)	Chi^2	t1/2 (min)	k_{on} (1/Ms)	k_{off} (1/s)	k_{on} (1/s)	k_{off} (1/s)	K_D (nM)	Chi^2	t1/2 (min)
V2 scDb	2.5×10^5	1.91×10^{-2}	1.85×10^{-3}	2.61×10^{-3}	34	10.7	0.6	Nd	Nd	Nd	Nd	NB	-	-
$F53W^{V2}$ scDb	1.66×10^5	1.5×10^{-2}	1.19×10^{-3}	1.59×10^{-4}	10.6	3.54	0.77	Nd	Nd	Nd	Nd	NB	-	-
$V104N^{V2}$ scDb	2.75×10^5	9.37×10^{-2}	9.57×10^{-4}	1.15×10^{-3}	185.5	1.37	0.12	Nd	Nd	Nd	Nd	NB	-	-
$V104R^{V2}$ scDb	1.32×10^5	0.93×10^{-2}	7.24×10^{-4}	7.47×10^{-5}	6.6	2.82	1.24	9.68×10^3	0.14	2.19×10^{-3}	2×10^{-3}	8904	0.117	0.08

	**$KRAS^{G12V}$-HLA-A*011:01**							**$KRAS^{WT}$-HLA-A*011:01**				
	$k_{on}1$ (1/Ms)	$k_{off}1$ (1/s)	$k_{on}2$ (1/s)	$k_{off}2$ (1/s)	K_D (uM)	Chi^2	t1/2 (min)	k_{on} (1/Ms)	k_{off} (1/s)	K_D (nM)	Chi^2	t1/2 (min)
V2 scDb	5.45×10^4	0.73	6.02×10^{-3}	0.05	12.1	0.046	0.01	Nd	Nd	NB	-	-
$F53W^{V2}$ scDb	Nd	Nd	Nd	Nd	NB	-		Nd	Nd	NB	-	-
$V104N^{V2}$ scDb	Nd	Nd	Nd	Nd	NB	-		Nd	Nd	NB	-	-
$V104R^{V2}$ scDb	9.69×10^3	0.12	1.89×10^{-3}	0.003	7.8	0.05	0.09	Nd	Nd	NB	-	-
