# Supplementary material for: Hydrophobic interactions dominate the recognition of a KRAS G12V neoantigen
Source: Nat Commun. 2023 Aug 21;14:5063. doi: 10.1038/s41467-023-40821-w (PMC10442379; doi:10.1038/s41467-023-40821-w)
Supplement: Supplementary file 3 — Description of Additional Supplementary Files [file 41467_2023_40821_MOESM3_ESM.pdf]

<u>Description of Additional Supplementary Files</u>

Supplementary Data 1: NGS Sequencing Data for V2 variant library. Variant frequency per site as determined by next generation sequencing was provided by Twist Bioscience after library synthesis. WT, wild type. The theoretical frequency for each variant is 5.26% (1/19).

Supplementary Movie 1: 3D variability analysis of the V2-Fab/KRAS^{G12V}-HLA-A*03:01 complex. Movie was captured over frames 0 to 19 emphasizing the flexibility of the constant regions of the V2-Fab.
