# Supplementary material for: Hydrophobic interactions dominate the recognition of a KRAS G12V neoantigen
Source: Nat Commun. 2023 Aug 21;14:5063. doi: 10.1038/s41467-023-40821-w (PMC10442379; doi:10.1038/s41467-023-40821-w)
Supplement: Supplementary file 6 — Reporting Summary [file 41467_2023_40821_MOESM6_ESM.pdf]

nature portfolio

Corresponding author(s): Sandra B. Gabelli, Shibin Zhou

Last updated by author(s): Aug 8, 2023

Reporting Summary

Nature Portfolio wishes to improve the reproducibility of the work that we publish. This form provides structure for consistency and transparency in reporting. For further information on Nature Portfolio policies, see our Editorial Policies and the Editorial Policy Checklist.

Statistics

For all statistical analyses, confirm that the following items are present in the figure legend, table legend, main text, or Methods section.

n/a	Confirmed	
☐	☒	The exact sample size (*n*) for each experimental group/condition, given as a discrete number and unit of measurement
☐	☒	A statement on whether measurements were taken from distinct samples or whether the same sample was measured repeatedly
☐	☒	The statistical test(s) used AND whether they are one- or two-sided<br>*Only common tests should be described solely by name; describe more complex techniques in the Methods section.*
☒	☐	A description of all covariates tested
☒	☐	A description of any assumptions or corrections, such as tests of normality and adjustment for multiple comparisons
☐	☒	A full description of the statistical parameters including central tendency (e.g. means) or other basic estimates (e.g. regression coefficient) AND variation (e.g. standard deviation) or associated estimates of uncertainty (e.g. confidence intervals)
☐	☒	For null hypothesis testing, the test statistic (e.g. *F*, *t*, *r*) with confidence intervals, effect sizes, degrees of freedom and *P* value noted<br>*Give P values as exact values whenever suitable.*
☒	☐	For Bayesian analysis, information on the choice of priors and Markov chain Monte Carlo settings
☒	☐	For hierarchical and complex designs, identification of the appropriate level for tests and full reporting of outcomes
☒	☐	Estimates of effect sizes (e.g. Cohen's *d*, Pearson's *r*), indicating how they were calculated

Our web collection on statistics for biologists contains articles on many of the points above.

Software and code

Policy information about availability of computer code

Data collection	LSDCGui (Life Science Data Collection software) version 1.0
Data analysis	coot 0.8<br>XDS Version January 26, 2018<br>PyMOL 2.4.2<br>CCP4 suite v7.0 (includes MolRep, PHASER, Refmac5, CONTACT)<br>Biacore Insight Evaluation Software<br>CryoSPARC v3.3.1<br>Relion 3.1<br>USCF ChimeraX-1.2.5<br>Phenix v1.18.2-3874-000<br>GROMACS 2022<br>CHARMM36m<br>GraphPad Prism 9.2.0

For manuscripts utilizing custom algorithms or software that are central to the research but not yet described in published literature, software must be made available to editors and reviewers. We strongly encourage code deposition in a community repository (e.g. GitHub). See the Nature Portfolio guidelines for submitting code & software for further information.

Data

Policy information about availability of data

All manuscripts must include a data availability statement. This statement should provide the following information, where applicable:

- Accession codes, unique identifiers, or web links for publicly available datasets
- A description of any restrictions on data availability
- For clinical datasets or third party data, please ensure that the statement adheres to our policy

The V2-IgG/KRASG12V-HLA-A*03:01 ternary complex data generated in this study have been deposited in the wwPBD database under accession code 7STF [https://doi.org/10.2210/pdb7STF/pdb] and Electron Microscopy Data Bank under accession code EMD-25427 [https://www.ebi.ac.uk/emdb/EMD-25427]. The KRASWT-HLA-A*03:01 complex data generated in this study have been deposited in the wwPDB database under accession code 8DVG [https://www.doi.org/10.2210/pdb8DVG/pdb]. The PDB ID 6O9B [https://doi.org/10.2210/pdb6O9B/pdb] was used as the search model for the KRASWT-HLA-A*03:01 structure determination. The coordinates of the KRASWT-HLA-A*03:01 monomer (determined in this paper) PDB ID 8DVG [https://www.rcsb.org/structure/unreleased/8DVG]) and PDB entry 7KGU [https://doi.org/10.2210/pdb7KGU/pdb] (2Q1-Fab) were used as search models for the V2-IgG/KRASG12V-HLA-A*03:01 complex. Source data are provided with this paper. The SDS-PAGE gels, SPR sensorgrams, co-culture experiments and DSF data generated in this study are provided in the Supplementary Information/Source Data file.

Human research participants

Policy information about studies involving human research participants and Sex and Gender in Research.

Reporting on sex and gender	Not Applicable
Population characteristics	Not Applicable
Recruitment	Not Applicable
Ethics oversight	Not Applicable

Note that full information on the approval of the study protocol must also be provided in the manuscript.

Field-specific reporting

Please select the one below that is the best fit for your research. If you are not sure, read the appropriate sections before making your selection.

☒ Life sciences ☐ Behavioural & social sciences ☐ Ecological, evolutionary & environmental sciences

For a reference copy of the document with all sections, see nature.com/documents/nr-reporting-summary-flat.pdf

Life sciences study design

All studies must disclose on these points even when the disclosure is negative.

Sample size	While no sample-size calculations were performed, non-structural data was obtained with either three or more biological or technical replicates which we deemed to be appropriate based on previous publications in the field.
Data exclusions	No data were excluded from the analyses.
Replication	The replication number is indicated in the figure legend of the corresponding figures where applicable. All attempts at replication were successful.
Randomization	Randomization was not relevant to this particular study. All experiments were performed in vitro with immunological and structural/molecular/cell biology techniques whereby the investigator both designs and performs said experiment.
Blinding	Blinding was not relevant to this study. All experiments were performed in vitro with immunological and structural/molecular/cell biology techniques whereby the investigator both designs and performs said experiment.

Reporting for specific materials, systems and methods

We require information from authors about some types of materials, experimental systems and methods used in many studies. Here, indicate whether each material, system or method listed is relevant to your study. If you are not sure if a list item applies to your research, read the appropriate section before selecting a response.

Materials & experimental systems

n/a	Involved in the study
☐	☒ Antibodies
☐	☒ Eukaryotic cell lines
☒	☐ Palaeontology and archaeology
☒	☐ Animals and other organisms
☒	☐ Clinical data
☒	☐ Dual use research of concern

Methods

n/a	Involved in the study
☒	☐ ChIP-seq
☒	☐ Flow cytometry
☒	☐ MRI-based neuroimaging

Antibodies

Antibodies used

- fd/M13 bacteriophage Antibody [polyclonal]: Novus Biologicals #NB100-1633
- Ultra-LEAF purified anti-human CD3 clone OKT3 (317347, Biolegend, San Diego, CA)
- goat anti-rabbit IgG (H+L) Secondary (HRP) antibody (NB7160, Novus, Centennial, CO)
- chicken anti-Protein L HRP antibody (ab63506, Abcam)

Validation

All antibodies used are well-validated and highly-cited. Please see below for links to the manufacturer's page for details with regards to their validation methodology and data, as well as relevant citations:
- fd/M13 bacteriophage Antibody [polyclonal]:
  --> https://www.novusbio.com/products/fd-m13-bacteriophage-antibody_nb100-1633
- goat anti-rabbit IgG (H+L) Secondary (HRP) antibody (NB7160, Novus, Centennial, CO)
  -->https://www.novusbio.com/products/igg-h-l-antibody_nb7160
- Ultra-LEAF purified anti-human CD3 clone OKT3 (317347, Biolegend, San Diego, CA)
  --> https://www.biolegend.com/en-us/products/ultra-leaf-purified-anti-human-cd3-antibody-7745?GroupID=BLG4203
- chicken anti-Protein L HRP antibody (ab63506, Abcam)
  --https://www.abcam.com/products/primary-antibodies/protein-l-antibody-hrp-ab63506.html

Eukaryotic cell lines

Policy information about cell lines and Sex and Gender in Research

Cell line source(s)

Jurkat (ATCC TIB-152), Raji (ATCC CCL-86), RPMI-6666 (ATCC CCL-113) and NCI-H358 (ATCC CRL-5807) cells were obtained from American Type Culture Collection (ATCC). T2A3 cells, an engineered cell line derivative of T2 cells, were a kind gift from Eric Lutz and Elizabeth Jaffee (JHU). HEK293FT (R70007) and Expi293F (A15427) cells were obtained from Thermo Fisher Scientific.

Authentication

Cell lines were not authenticated.

Mycoplasma contamination

All cell lines tested negative for mycoplasma contamination.

Commonly misidentified lines
(See ICLAC register)

No commonly misidentified lines were employed in this study.
